# Impact of Long SARS-CoV-2 Omicron Infection on the Health Care Burden: Comparative Case-Control Study Between Omicron and Pre-Omicron Waves

**DOI:** 10.2196/53580

**Published:** 2024-09-03

**Authors:** Bernardo Valdivieso-Martinez, Victoria Lopez-Sanchez, Inma Sauri, Javier Diaz, Jose Miguel Calderon, Maria Eugenia Gas-Lopez, Laura Lidon, Juliette Philibert, Jose Luis Lopez-Hontangas, David Navarro, Llanos Cuenca, Maria Jose Forner, Josep Redon

**Affiliations:** 1 Medical Research Institute, Hospital Universitari i Politècnic La Fe Valencia Spain; 2 Hospital Clínico de la Comunidad Valenciana (INCLIVA) Research Institute University of Valencia Valencia Spain; 3 Hospital Clinico of Valencia Valencia Spain; 4 Universitat Politècnica de València Valencia Spain

**Keywords:** Omicron, long COVID, post–COVID-19, diagnostics, primary care, specialist, emergency department, hospitalization

## Abstract

**Background:**

Following the initial acute phase of COVID-19, health care resource use has escalated among individuals with SARS-CoV-2 infection.

**Objective:**

This study aimed to compare new diagnoses of long COVID and the demand for health services in the general population after the Omicron wave with those observed during the pre-Omicron waves, using similar assessment protocols for both periods and to analyze the influence of vaccination.

**Methods:**

This matched retrospective case-control study included patients of both sexes diagnosed with acute SARS-CoV-2 infection using reverse transcription polymerase chain reaction or antigen tests in the hospital microbiology laboratory during the pandemic period regardless of whether the patients were hospitalized. We included patients of all ages from 2 health care departments that cover 604,000 subjects. The population was stratified into 2 groups, youths (<18 years) and adults (≥18 years). Patients were followed-up for 6 months after SARS-CoV-2 infection. Previous vaccination, new diagnoses, and the use of health care resources were recorded. Patients were compared with controls selected using a prospective score matched for age, sex, and the Charlson index.

**Results:**

A total of 41,577 patients with a history of prior COVID-19 infection were included, alongside an equivalent number of controls. This cohort encompassed 33,249 (80%) adults aged ≥18 years and 8328 (20%) youths aged <18 years. Our analysis identified 40 new diagnoses during the observation period. The incidence rate per 100 patients over a 6-month period was 27.2 for vaccinated and 25.1 for unvaccinated adults (*P*=.09), while among youths, the corresponding rates were 25.7 for vaccinated and 36.7 for unvaccinated individuals (*P*<.001). Overall, the incidence of new diagnoses was notably higher in patients compared to matched controls. Additionally, vaccinated patients exhibited a reduced incidence of new diagnoses, particularly among women (*P*<.001) and younger patients (*P*<.001) irrespective of the number of vaccine doses administered and the duration since the last dose. Furthermore, an increase in the use of health care resources was observed in both adult and youth groups, albeit with lower figures noted in vaccinated individuals. In the comparative analysis between the pre-Omicron and Omicron waves, the incidence of new diagnoses was higher in the former; however, distinct patterns of diagnosis were evident. Specifically, depressed mood (*P*=.03), anosmia (*P*=.003), hair loss (*P*<.001), dyspnea (<0.001), chest pain (*P*=.04), dysmenorrhea (*P*<.001), myalgia (*P*=.011), weakness (*P*<.001), and tachycardia (*P*=.015) were more common in the pre-Omicron period. Similarly, health care resource use, encompassing primary care, specialist, and emergency services, was more pronounced in the pre-Omicron wave.

**Conclusions:**

The rise in new diagnoses following SARS-CoV-2 infection warrants attention due to its potential implications for health systems, which may necessitate the allocation of supplementary resources. The absence of vaccination protection presents a challenge to the health care system.

## Introduction

The all-cause disease burden worldwide in 2020 and 2021 was the highest in the past 3 decades, mainly due to the consequences of the SARS-CoV-2 pandemic [1]. In fact, the worldwide population was profoundly affected not only by the acute infection, resulting in increased morbidity and mortality [2], but also by persistent symptoms after the initial acute phase of illness or the impact on organ systems with the emergence of new diseases [3-12]. As a result, health care resource use has escalated among individuals with SARS-CoV-2 infection. Ongoing viral mutations and the introduction of vaccines may result in varying degrees of impact, and periodic assessment of the disease burden may help to better define strategies to mitigate the impact.

Rapid dissemination of various SARS-CoV-2 variants and the introduction of vaccines have raised expectations of altered impacts on long COVID. Subsequent to the prevalence of the Alpha and Delta variants during the initial pandemic waves in the pre-Omicron period, SARS-CoV-2 Omicron (PANGO B.1.1.529) swiftly propagated across Europe from December 2021 to February 2022. Clinically, the Omicron variant induced less severe acute illness than its predecessors, and certain studies reported a reduced incidence of postacute-phase impacts [13-19], both in adults and in children [20-24]. Nevertheless, conflicting data have surfaced in the literature [25], and the potential influence of prior vaccination remains uncertain [26-28]. Evaluating the genuine repercussions of SARS-CoV-2 infection in the realm of long COVID following the acute phase proves intricate due to myriad factors. Discrepancies in defining criteria, observation durations, encompassed medical conditions, and symptom dynamics may account for disparities among reported studies. Assessing the tangible effect on the health care resource burden following the acute infection episode holds paramount significance, given its implications for the resources that health care systems must allocate. One effective approach to gauge this impact is to leverage the data available in electronic health records (EHRs), which serve as a valuable source of information regarding the health care resource demands placed on health systems by encompassing a wealth of data on diagnoses, medication usage, and health care resource requirements [29].

Based on the EHRs of 2 health care departments (HCDs) in the Valencian Community of more than 600,000 inhabitants, this study aimed to compare new diagnoses of long COVID and the demand for health services in the general population after the Omicron variant wave with those observed during the pre-Omicron Alpha and Delta waves using similar assessment protocols for both periods. In addition, it sought to elucidate the potential influence of vaccination in mitigating these effects in different age groups.

## Methods

### Study Design and Participants

A case-control study with retrospective observation of health care data collected from ABUCASIS, the EHR of the Valencia Community was conducted. Administrative data, diagnoses, all prescriptions and dispensations of subsidized treatments, and the use of health services are linked in a database that integrates all health care interventions. In this study, the data included observations of patients with SARS-CoV-2 infection from a total of 604,000 subjects from 2 different HCDs (centers A and B).

### Ethical Considerations

Exemption from obtaining informed consent was permissible based on the 17th additional provision of Spanish Organic Law 3/2018, dated December 5, which pertains to the protection of personal data and the guarantee of digital rights. This provision legalizes the use of pseudonymized personal data for health-related purposes, particularly in the realm of biomedical research. To harness pseudonymized personal data for the objectives of public health and biomedical research, the following criteria were met: A clear demarcation in terms of both technical and functional aspects was maintained between the research team and individuals responsible for executing the pseudonymization process, as well as safeguarding the information that could potentially facilitate reidentification. Data collection and analysis were carried out considering the protection of patients’ privacy by means of a 2-layered method of pseudo anonymization, and the information was managed as aggregated data. Access to pseudonymized data was only permitted for the research team under specific conditions:

- An explicit commitment to maintain confidentiality and abstain from any reidentification endeavors was established.
- Stringent security measures were instituted to forestall reidentification and unauthorized access by third parties.

The research was conducted in full compliance with the provisions of Regulation (EU) 2016/679 of the European Parliament and of the Council of April 27, 2016, on the protection of individuals with regard to the processing of personal data and on the free movement of such data. The study also complied with the 17th additional provision of Spanish Organic Law 3/2018 of December 5, the corresponding European norms (General Data Protection Regulation [GDPR]) [30], and the applicable sectoral legislation. The information was available for research and pseudo anonymization in accordance with the Spanish Data Protection Act, and the study was approved by the Ethics and Clinical Trials Committee of the Hospital Clinico of Valencia and the Hospital Universitari i Politècnic La Fe of Valencia.

### Subjects and Procedures

Cases included patients of both sexes diagnosed with acute SARS-CoV-2 infection using reverse transcription polymerase chain reaction (RT-PCR) or antigen tests in the hospital microbiology laboratory during the SARS-CoV-2 Omicron pandemic period regardless of whether the patients were hospitalized. The population was stratified into 2 groups, youths under the age of 18 years and adults aged ≥18 years.

**Figure 1:**
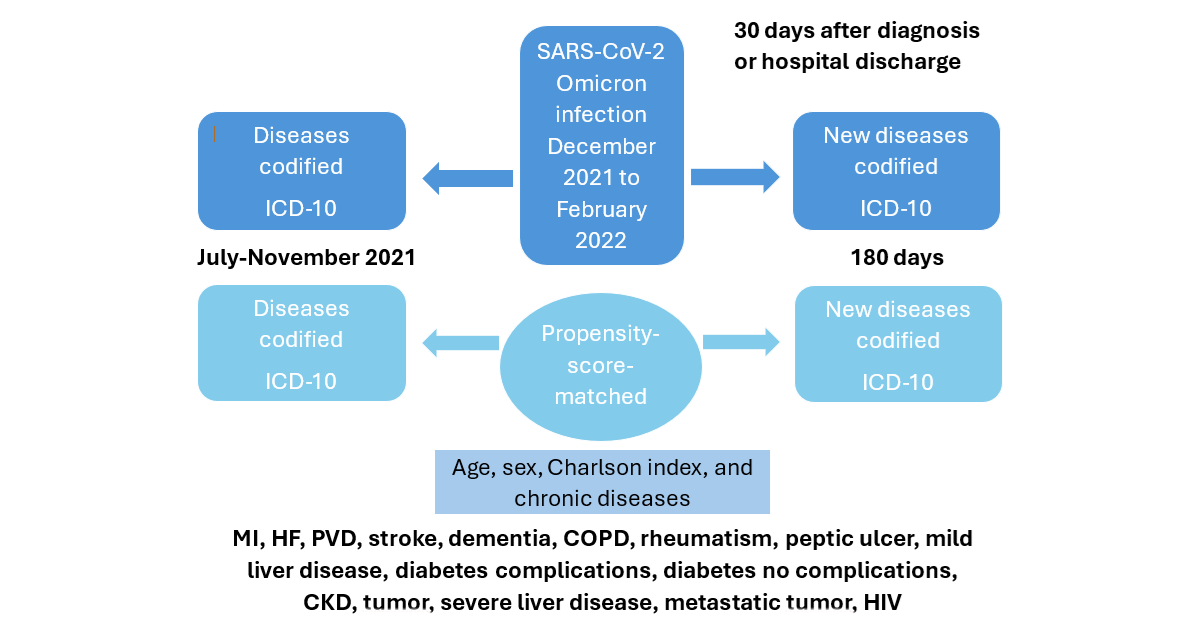
Study design. Cases included patients of both sexes diagnosed with acute SARS-CoV-2 infection, and the same number of matched controls without infection were selected using a propensity score matched for age, sex, previous illnesses, and the same time period as the case index. The observation period for new diagnoses and health care resource use was 6 months after 30 days from virus diagnosis or hospital discharge. CKD: chronic kidney disease; COPD: chronic obstructive pulmonary disease; HF: heart failure; ICD-10: International Classification of Diseases, 10th Revision; MI: myocardial infarction; PVD: pulmonary vascular disease.

The study design has been previously detailed [31], with a summary provided in Figure 1. In brief, the cases of Omicron infection included individuals with acute infection diagnosed between December 1, 2021, and February 28, 2022. The observation period for the long COVID phase commenced 30 days following the date of diagnosis, either in primary care or after hospital discharge, and extended for up to 6 months, totaling 180 days. During this period, newly occurring diseases and medications, not present prior to the infection, were meticulously documented within the EHR system. Vaccination details, including the number of doses administered and the time elapsed since the last dose before infection, were also ascertained. Throughout the observation period, the collection of data encompassed the identification of newly diagnosed conditions and prescriptions. Additionally, the use of health care resources, comprising the number of patients and visits to primary care physicians, specialists, emergency rooms, and hospitalization, was extracted from administrative records sourced from primary care health care centers, hospital outpatient clinics, emergency departments, and hospitalization units.

The analysis of new diagnoses included an equal number of matched controls who had not been infected with SARS-CoV-2. The control group was generated through propensity score matching (PSM) that incorporated variables such as age, sex, the Charlson index, all preexisting chronic diseases (chronic conditions present prior to the pandemic period, including but not limited to myocardial infarction, heart failure, peripheral artery disease, stroke, dementia, chronic obstructive pulmonary disease, rheumatism, peptic ulcer, liver disease, diabetes, chronic kidney disease, tumor, metastatic tumor, and HIV infection), and the corresponding case’s time frame. Data from a prior study conducted using the same methodology during the pre-Omicron period, spanning from March to December 2020, were used to facilitate comparisons regarding the potential impact of Omicron [31].

### Statistical Analysis

Data were presented in the form of absolute numbers, incidences per 100 patients per 6 months, and percentages, where applicable. To assess differences in the incidence of new diagnoses among patients with Omicron infection, vaccinated individuals, and unvaccinated patients, as well as in comparison to the pre-Omicron period, statistical analyses were conducted using unpaired Student *t* tests and chi-squared tests. The potential long-term effects of vaccination, including the number of vaccine doses and the time elapsed since the last dose to the time of infection, were evaluated through logistic regression analysis, adjusted for potential confounding factors, such as age and sex. Furthermore, sensitivity analyses were performed by separately analyzing data from the 2 HCDs and comparing the results between them. All statistical analyses were carried out using R version 6.3.1 (R Foundation for Statistical Computing).

## Results

### General Characteristics of the Study Population

During the study period corresponding to the Omicron wave, a total of 41,577 patients were diagnosed with positive SARS-CoV-2 using RT-PCR or antigen tests. This patient cohort comprised 33,249 (80%) adults aged ≥18 years and 8328 (20%) youths aged <18 years. Prior to the onset of acute infection, 30,199 (90.8%) adults and 4417 (53%) youths had received the vaccine. The general characteristics of the study population are detailed in Table 1 for adults and in Table 2 for youths. Follow-up of the patients extended from 177 to 179 days after 30 days of confirmed infection. Among them, a total of 833 (2%) patients were hospitalized for acute SARS-CoV-2 Omicron infection, including 662 (79.5%) adults and 75 (9%) youths, with 24 (32%) of the latter being vaccinated.

**Table 1:** General characteristics of adult (≥18 years) study population cases in the 2 HCDs^a^ during Omicron and pre-Omicron waves.

Characteristic		Omicron (previous vaccine)			Omicron (no previous vaccine)			Pre-Omicron	
		Center A (n=17,441)	Center B (n=12,758)	Center A (n=1040)		Center B (n=2010)	Center A (n=16,382)		Center B (n=14,199)
Age (years), mean (SD)		46.16 (16.37)	48.78 (18.50)	41.75 (14.81)		40.87 (13.71)	47.63 (17.73)		45.93 (17.10)
Female, n (%)		9832 (56.4)	7110 (55.7)	570 (54.8)		1168 (58.1)	8861 (54.1)		7671 (54.0)
Days of observation, mean (SD)		179 (10.93)	179.58 (6.80)	177.98 (16.04)		177.11 (20.15)	176.06 (20.53)		179.00 (7.68)
Patients hospitalized, n (%)		458 (2.6)	204 (1.6)	78 (7.5)		68 (3.4)	1726 (10.5)		1357 (9.6)
First vaccination, date		June 6, 2021 10089 (57.95)	January 7, 2021 6668 (52.27)	N/A^b^		N/A	N/A		N/A
Patients with new diagnoses, n (%)		3535 (20.3)	2729 (21.4)	223 (21.4)		392 (19.5)	3082 (18.8)		2883 (20.3)
	1	2815 (16.1)	2128 (16.7)	171 (16.4)		293 (14.6)	2463 (15.0)		2182 (15.4)
	2	574 (3.3)	474 (3.7)	42 (3.9)		71 (3.5)	506 (3.1)		544 (3.8)
	3	121 (0.7)	101 (0.8)	8 (0.8)		20 (1.0)	87 (0.5)		116 (0.8)
	>3	25 (0.1)	26 (0.2)	2 (0.2)		8 (0.4)	26 (0.2)		41 (0.3)

^a^HCD: health care department.

^b^N/A: not applicable.

**Table 2:** General characteristics of youth (<18 years) study population cases in the 2 HCDs^a^ during Omicron and pre-Omicron waves.

Characteristic		Omicron (previous vaccine)			Omicron (no previous vaccine)			Pre-Omicron	
		Center A (n=2714)	Center B (n=2243)	Center A (n=1824)		Center B (n=1547)	Center A (n=3981)		Center B (n=3605)
Age (years), mean (SD)		12.58 (3.41)	12.17 (3.54)	5.31 (3.87)		5.91 (4.30)	10.23 (4.99)		9.42 (5.19)
Female, n (%)		1377 (50.7)	1123 (50.1)	864 (47.4)		719 (46.5)	1956 (49.1)		1783 (49.0)
Days of observation, mean (SD)		180 (0)	179.91 (3.76)	180 (0)		179.40 (9.01)	179.11 (4.21)		179.26 (3.89)
Patients hospitalized, n (%)		12 (0.4)	12 (0.5)	19 (1.0)		32 (2.1)	23 (0.6)		27 (0.7)
First vaccination, date		October 21, 2021 2243 (82.66)	June 12, 2021 1987 (88.60)	N/A^b^		N/A	N/A		N/A
Patients with new diagnoses, n (%)		511 (18.8)	421 (18.8)	499 (27.4)		404 (26.1)	660 (16.6)		584 (16.2)
	1	418 (15.4)	339 (15.1)	387 (21.2)		318 (20.6)	544 (13.7)		463 (12.8)
	2	84 (3.1)	70 (3.1)	97 (5.3)		70 (4.5)	95 (2.4)		90 (2.5)
	3	8 (0.3)	10 (0.4)	11 (0.6)		13 (0.8)	18 (0.5)		27 (0.7)
	>3	1 (0)	2 (0.1)	4 (0.2)		3 (0.2)	3 (0.1)		4 (0.1)

^a^HCD: health care department.

^b^N/A: not applicable.

### New Diagnostics

The new diagnoses observed during the follow-up period in patients with SARS-CoV-2 and Alpha and Omicron infections and in controls, regardless of their vaccination status, and the corresponding distribution of the *International Classification of Diseases, 10th Revision* (ICD-10) codes across affected systems are presented in Tables S1-S4 in Multimedia Appendix 1. Among adults, the number of new diagnoses for vaccinated and unvaccinated patients stood at 6642 and 636, respectively, with incidences per 100 patients over 6 months of 27.2 and 25.1, respectively (*P*=.09). In the case of youths, these numbers were lower, with 1014 diagnoses in vaccinated individuals and 967 in unvaccinated individuals, resulting in incidences of 25.7 and 36.7, respectively (*P*<.001). In adults, the vaccination status did not appear to significantly influence the number of new diagnoses per patient (*P*=.09), as indicated by the following percentages: vaccinated (n=4943, 16.4%, had 1 new diagnosis; n=1048, 3.5%, had 2 new diagnoses; n=222, 0.7%, had 3 new diagnoses; and n=51, 0.1%, had more than 3 new diagnoses) and unvaccinated (n=464, 15.5%, had only 1 new diagnosis; n=113, 3.7%, had 2 new diagnoses; n=28, 0.9%, had 3 new diagnoses; and n=10, 0.3%, had more than 3 new diagnoses), as shown in Table 1. Conversely, among youths, a notably higher number of events were observed among the unvaccinated group, with percentages as follows (*P*<.001): vaccinated (n=757, 15.2%, had 1 new diagnosis; n=154, 3.1%, had 2 new diagnoses; n=18, 0.4%, had 3 new diagnoses; and n=3, 0.1%, had more than 3 new diagnoses in center B) and unvaccinated (n=705, 20.9%, had 1 new diagnosis; n=167, 5%, had 2 new diagnoses; n=24, 0.7%, had 3 new diagnoses; and n=7, 0.2%, had more than 3 new diagnoses), as shown in Table 2. Ultimately, logistic regression analysis revealed a reduced risk of new diagnoses in women (0.39, 95% CI 0.35-0.43, *P*<.001) and younger patients (0.98, 95% CI 0.98-0.99, *P*<.001). However, the number of vaccine doses (0.98, 95% CI 0.86-1.11, *P*=.73) and the time elapsed from the last vaccine dose to infection (1.01, 95% CI 0.99-1.04, *P*=.42) were not found to be associated with the risk of new diagnoses. Diseases across systems, along with the number and incidence of each disease, are presented in Tables S1 and S2 in Multimedia Appendix 1 for adults and youths, respectively. Incidence data are further detailed in Tables S3 and S4 in Multimedia Appendix 1 for adults and youths, respectively. In the adult population, the most frequently occurring new diagnoses spanned neurophysical, infectious, digestive, respiratory, and musculoskeletal categories. Among the most common diseases were the following: (1) neurophysical conditions included anxiety, insomnia, headache, dizziness, and vertigo; (2) infectious diseases included acute pharyngitis and tonsillitis; (3) digestive conditions included functional dyspepsia, diarrhea, and abdominal pain; (4) respiratory issues included cough; and (5) musculoskeletal ailments included low back pain and weakness. In youths, acute pharyngitis was the most prevalent condition, with no notable difference between vaccinated and unvaccinated individuals.

When comparing the incidence of each new disease between vaccinated and unvaccinated individuals, significant differences were observed in adults for dizziness and giddiness (*P*=.01) and functional dyspepsia (*P*=.005). In contrast, among youths, significant differences were found for anxiety disorder (*P*<.001); dizziness and giddiness (*P*<.001); headache (*P*=.025); acute pharyngitis, tonsillitis, and fever (*P*<.001 for each); dermatitis, unspecified (*P*=.024); cough (*P*<.001); functional dyspepsia (*P*=.009); dysmenorrhea (*P*=.001); low back pain (*P*=.004); weakness (*P*=.033); conjunctivitis (*P*<.001); and recurrent oral aphthae (*P*<.001). Further details regarding the statistical significance of each new diagnosis are provided in Table S5 in Multimedia Appendix 1.

### Health Care Resources

The use of health care services, encompassing primary care, specialist consultations, emergency visits, and hospital admissions, among both vaccinated and unvaccinated cases and controls in the adult and youth populations is detailed in Tables 3 and 4, respectively. In vaccinated adults, patients exhibited higher health care service use compared to controls (Table 3). This increase was observed across primary care (n=33,672, 25%, more visits in n=7575, 15%, more patients, *P*<.001), specialist consultations (n=6747, 19%, more visits in n=1875, 17%, more patients, *P*<.001), and emergency admissions (n=1673, 25%, more visits in n=1026, 23%, more patients, *P*<.001). The escalation in resource use was even more pronounced in the unvaccinated population, with primary care visits seeing a 53% (n=6250) increase in visits among 46% (n=958) more patients (*P*<.001), specialist consultations witnessing a 49% (n=1527) surge in visits among 44% (n=413) more patients (*P*<.001), and emergency services experiencing a 27% (n=177) rise in visits among 19% (n=78) more patients (*P*=.002). In vaccinated youths, health care service use was also greater among patients with infection compared to controls, although the differences were smaller than in adults (Table 4). Specifically, there was a 24% (n=4348) increase in primary care visits among 13% (n=1501) more patients (*P*<.001), a 16% (n=417) uptick in specialist consultations among 12% (n=226) more patients (*P*<.001), and a 24% (n=240) rise in emergency visits among 20% (n=137) more patients (*P*<.001). Among unvaccinated youths, health care service use was likewise higher among patients with infection compared to controls, with differences again being smaller than in adults: primary care visits increased by 30% (n=5612) among 20% (n=571) more patients (*P*<.001), specialist consultations surged by 32% (n=667) among 30% (n=262) more patients (*P*<.001), and emergency visits saw a 32% (n=449) rise among 23% (n=190) more patients (*P*<.001).

Overall, hospital admissions were influenced by vaccination status in both adults (*P*<.001) and youths (*P*=.004), with higher demands observed among unvaccinated patients. Among adults, compared to controls, there were 23% (n=292) more patients in the vaccinated group and 48% (n=63) more patients in the unvaccinated group (*P*<.001). Among youths, there were 12% (n=9) more patients in the vaccinated group and 41% (n=44) more patients in the unvaccinated group (*P*<.001 for unvaccinated, *P*=.55 for vaccinated).

**Table 3:** Burden of health care resources (primary care, specialist consultations, emergency room, hospital admissions, and CCU^a^ admissions) in the adult study population in the 2 HCDs^b^ during the 2 study periods.^c^

Health care resource			Omicron (previous vaccine)			Omicron (no previous vaccine)				Pre-Omicron		
			Center A	Center B		Center A		Center B	Center A			Center B
**Primary care visits**												
	Visits	Cases: 76,957 Controls: 56,503 Difference: 20,454 (27%)		Cases: 56,855 Controls: 43,637 Difference: 13,218 (23%)	Cases: 4050 Controls: 1887 Difference: 2163 (53%)		Cases: 7701 Controls: 3614 Difference: 4087 (53%)		Cases: 94,131 Controls: 76,702 Difference: 17,429 (19%)		Cases: 76,902 Controls: 56,563 Difference: 20,339 (26%)	
	Patients	Cases: 13,360 Controls: 11,346 Difference: 6101 (15%)		Cases: 9893 Controls: 8419 Difference: 1474 (15%)	Cases: 735 Controls: 401 Difference: 334 (45%)		Cases: 1347 Controls: 723 Difference: 624 (46%)		Cases: 14,074 Controls: 12,967 Difference: 1107 (8%)		Cases: 11,896 Controls: 9905 Difference: 1991 (17%)	
**Specialist visits**												
	Visits	Cases: 19,969 Controls: 15,472 Difference: 4497 (23%)		Cases: 15,768 Controls: 13,518 Difference: 2250 (14%)	Cases: 1063 Controls: 542 Difference: 521 (49%)		Cases: 2054 Controls: 1048 Difference: 1006 (49%)		Cases: 21,439 Controls: 16,255 Difference: 5184 (24%)		Cases: 19,892 Controls: 13,540 Difference: 6352 (32%)	
	Patients	Cases: 6298 Controls: 5200 Difference: 1098 (17%)		Cases: 4728 Controls: 3951 Difference: 777 (16%)	Cases: 356 Controls: 167 Difference: 189 (53%)		Cases: 584 Controls: 360 Difference: 224 (38%)		Cases: 6389 Controls: 5106 Difference: 1283 (20%)		Cases: 5331 Controls: 3720 Difference: 1611 (30%)	
**Emergency room visits**												
	Visits	Cases: 4081 Controls: 3018 Difference: 1063 (26%)		Cases: 2480 Controls: 1870 Difference: 610 (25%)	Cases: 265 Controls: 169 Difference: 96 (36%)		Cases: 389 Controls: 308 Difference: 81 (21%)		Cases: 3503 Controls: 2746 Difference: 1665 (22%)		Cases: 2727 Controls: 1706 Difference: 1021 (37%)	
	Patients	Cases: 2682 Controls: 2060 Difference: 622 (23%)		Cases: 1736 Controls: 1332 Difference: 404 (23%)	Cases: 159 Controls: 95 Difference: 64 (40%)		Cases: 247 Controls: 233 Difference: 14 (6%)		Cases: 2270 Controls: 1838 Difference: 432 (19%)		Cases: 1885 Controls: 1227 Difference: 658 (35%)	
**Hospital admissions**												
	Admissions	Cases: 850 Controls: 656 Difference: 194 (23%)		Cases: 472 Controls: 374 Difference: 98 (21%)	Cases: 43 Controls: 20 Difference: 23 (53%)		Cases: 88 Controls: 48 Difference: 40 (45%)		Cases: 838 Controls: 792 Difference: 46 (5%)		Cases: 603 Controls: 438 Difference: 165 (27%)	
	Admissions from emergency	Cases: 303 Controls: 307 Difference: –4 (–1%)		Cases: 181 Controls: 118 Difference: 63 (35%)	Cases: 18 Controls: 10 Difference: 8 (44%)		Cases: 42 Controls: 15 Difference: 27 (64%)		Cases: 417 Controls: 476 Difference: –59 (–14%)		Cases: 318 Controls: 208 Difference: 110 (35%)	
	Admissions scheduled	Cases: 246 Controls: 160 Difference: 86 (35%)		Cases: 159 Controls: 136 Difference: 23 (14%)	Cases: 10 Controls: 5 Difference: 5 (50%)		Cases: 21 Controls: 21 Difference: 0		Cases: 196 Controls: 160 Difference: 36 (18%)		Cases: 110 Controls: 96 Difference: 14 (13%)	
	Patients	Cases: 741 Controls: 555 Difference: 186 (25%)		Cases: 436 Controls: 352 Difference: 84 (19%)	Cases: 39 Controls: 18 Difference: 21 (54%)		Cases: 82 Controls: 45 Difference: 37 (45%)		Cases: 672 Controls: 641 Difference: 31 (5%)		Cases: 518 Controls: 366 Difference: 152 (29%)	
**CCU admissions**												
	Admissions	Cases: 9 Controls: 15 Difference: –6 (–67%)		Cases: 22 Controls: 11 Difference: 11 (50%)	Cases: 1 Controls: 0 Difference: 1 (100%)		Cases: 22 Controls: 11 Difference: 11 (50%)		Cases: 17 Controls: 36 Difference: 19 (–112%)		Cases: 15 Controls: 19 Difference: –4 (–27%)	
	Patients	Cases: 7 Controls: 13 Difference: –6 (–86%)		Cases: 20 Controls: 10 Difference: 10 (50%)	Cases: 1 Controls: 0 Difference: 1 (100%)		Cases: 20 Controls: 10 Difference: 10 (50%)		Cases: 15 Controls: 33 Difference: –18 (–120%)		Cases: 14 Controls: 17 Difference: –3 (–21%)	

^a^CCU: critical care unit.

^b^HCD: health care department.

^c^Data show the number of cases and controls. The difference is presented as both a numerical value (n) and a percentage calculated as ([cases – controls]/cases) × 100.

**Table 4:** Burden of health care resources (primary care, specialist consultations, emergency room, hospital admissions, and CCU^a^ admissions) in the adult study population in the 2 HCDs^b^ during the 2 study periods.^c^

Health care resource			Omicron (previous vaccine)			Omicron (no previous vaccine)				Pre-Omicron		
			Center A	Center B		Center A		Center B	Center A			Center B
**Primary care visits**												
	Visits	Cases: 9525 Controls: 6808 Difference: 2717 (29%)		Cases: 8578 Controls: 6947 Difference: 1631 (19%)	Cases: 10,595 Controls: 7344 Difference: 3251 (31%)		Cases: 8375 Controls: 6014 Difference: 2361 (28%)		Cases: 14,801 Controls: 13,045 Difference: 1756 (12%)		Cases: 14,195 Controls: 9769 Difference: 4426 (31%)	
	Patients	Cases: 2094 Controls: 1758 Difference: 1336 (16%)		Cases: 1814 Controls: 1649 Difference: 165 (9%)	Cases: 1519 Controls: 1248 Difference: 271 (18%)		Cases: 1303 Controls: 1003 Difference: 300 (23%)		Cases: 2988 Controls: 2681 Difference: 307 (10%)		Cases: 2665 Controls: 1952 Difference: 713 (27%)	
**Specialist visits**												
	Visits	Cases: 1714 Controls: 1442 Difference: 272 (16%)		Cases: 1624 Controls: 1479 Difference: 145 (9%)	Cases: 1066 Controls: 661 Difference: 405 (38%)		Cases: 988 Controls: 726 Difference: 262 (27%)		Cases: 2493 Controls: 2065 Difference: 428 (17%)		Cases: 2243 Controls: 1710 Difference: 533 (24%)	
	Patients	Cases: 768 Controls: 622 Difference: 146 (19%)		Cases: 649 Controls: 569 Difference: 80 (12%)	Cases: 458 Controls: 314 Difference: 144 (31%)		Cases: 409 Controls: 291 Difference: 118 (29%)		Cases: 1008 Controls: 885 Difference: 123 (12%)		Cases: 847 Controls: 633 Difference: 214 (25%)	
**Emergency room visits**												
	Visits	Cases: 550 Controls: 400 Difference: 150 (27%)		Cases: 439 Controls: 349 Difference: 90 (21%)	Cases: 796 Controls: 521 Difference: 275 (35%)		Cases: 612 Controls: 439 Difference: 173 (28%)		Cases: 675 Controls: 486 Difference: 189 (28%)		Cases: 610 Controls: 418 Difference: 192 (31%)	
	Patients	Cases: 390 Controls: 300 Difference: 90 (23%)		Cases: 311 Controls: 264 Difference: 47 (15%)	Cases: 479 Controls: 332 Difference: 147 (31%)		Cases: 361 Controls: 318 Difference: 43 (12%)		Cases: 468 Controls: 367 Difference: 101 (22%)		Cases: 431 Controls: 315 Difference: 116 (27%)	
**Hospital admissions**												
	Admissions	Cases: 32 Controls: 25 Difference: 9 (22%)		Cases: 39 Controls: 52 Difference: –13 (–33%)	Cases: 44 Controls: 23 Difference: 21 (48%)		Cases: 55 Controls: 32 Difference: 23 (42%)		Cases: 62 Controls: 32 Difference: 30 (48%)		Cases: 84 Controls: 57 Difference: 27 (32%)	
	Admissions from emergency	Cases: 15 Controls: 14 Difference: 1 (7%)		Cases: 11 Controls: 14 Difference: –3 (–27%)	Cases: 20 Controls: 10 Difference: 10 (50%)		Cases: 19 Controls: 19 Difference: 0		Cases: 24 Controls: 7 Difference: 17 (71%)		Cases: 39 Controls: 21 Difference: 18 (46%)	
	Admissions scheduled	Cases: 5 Controls: 2 Difference: 3 (60%)		Cases: 17 Controls: 19 Difference: –2 (–12%)	Cases: 7 Controls: 6 Difference: 1 (14%)		Cases: 23 Controls: 10 Difference: 13 (57%)		Cases: 17 Controls: 4 Difference: 13 (76%)		Cases: 26 Controls: 24 Difference: 2 (8%)	
	Patients	Cases: 31 Controls: 23 Difference: 8 (26%)		Cases: 36 Controls: 52 Difference: –16 (–44%)	Cases: 39 Controls: 22 Difference: 17 (44%)		Cases: 51 Controls: 31 Difference: 20 (39%)		Cases: 51 Controls: 31 Difference: 20 (39%)		Cases: 72 Controls: 49 Difference: 23 (32%)	
**CCU admissions**												
	Admissions	Cases: 0 Controls: 0 Difference: 0		Cases: 2 Controls: 1 Difference: 1 (50%)	Cases: 0 Controls: 0 Difference: 0		Cases: 2 Controls: 1 Difference: 1 (50%)		Cases: 0 Controls: 0 Difference: 0		Cases: 1 Controls: 5 Difference: –4 (–400%)	
	Patients	Cases: 0 Controls: 0 Difference: 0		Cases: 2 Controls: 1 Difference: 1 (50%)	Cases: 0 Controls: 0 Difference: 0		Cases: 2 Controls: 1 Difference: 1 (50%)		Cases: 0 Controls: 0 Difference: 0		Cases: 1 Controls: 4 Difference: –3 (–300%)	

^a^CCU: critical care unit.

^b^HCD: health care department.

^c^Data show the number of cases and controls. The difference is presented as both a numerical value (n) and a percentage calculated as ([cases – controls]/cases) × 100.

### Comparison With the Data From Pre-Omicron SARS-CoV-2 Waves

The general characteristics of the study population, along with the occurrence of new diagnoses and the health care burden associated with Omicron and pre-Omicron waves, are presented in Tables 3 and 4 for adults and youths, respectively. In adults, the prevalence of certain diagnoses in the pre-Omicron period was higher than in the Omicron period. Specifically, depressed mood (*P*=.03), anosmia (*P*=.003), hair loss (*P*<.001), dyspnea (*P*<.001), chest pain (*P*=.04), dysmenorrhea (*P*<.001), myalgia (*P*=.011), weakness (*P*<.001), and tachycardia (*P*=.015) were more common in the pre-Omicron period. Conversely, cough (*P*<.001), diarrhea (*P*=.03), low back pain (*P*<.001), and conjunctivitis (*P*<.001) were more prevalent in the Omicron period. In youths, anosmia (*P*=.003) was found to be more common in the pre-Omicron period. Despite these differences in frequency, the burden of long COVID complaints on health care services was similar between the Omicron and pre-Omicron periods, with the exception of musculoskeletal complaints.

Overall, the use of health care resources was higher during the pre-Omicron period in both adults (primary care, *P*<.001; specialist, *P*<.001; emergency, *P*<.001) and youths (primary care, *P*<.001; specialist, *P*<.001; emergency, <.001). Furthermore, the demand for health care services was greater among the unvaccinated population in the Omicron period than in the pre-Omicron period.

### Sensitivity Analysis

**Figure 2:**
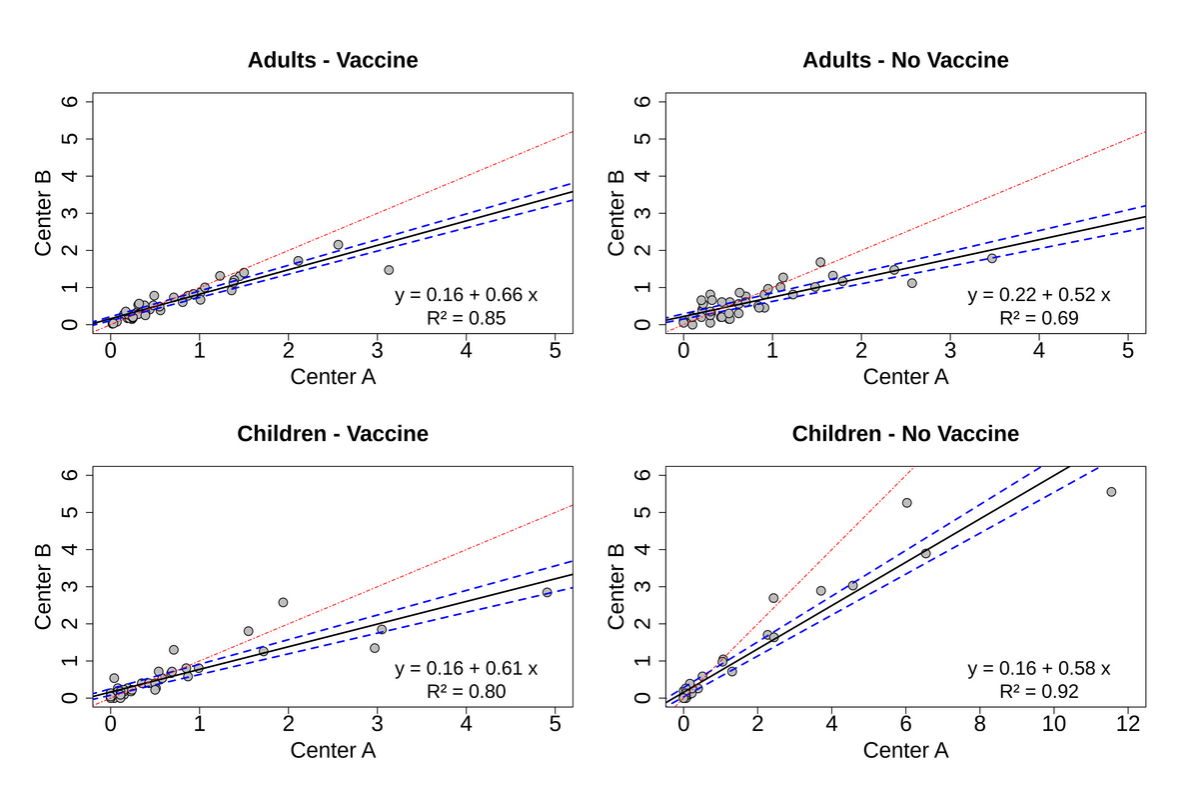
Correlation of new diagnoses between 2 health care areas in the study populations aged ≥18 and <18 years old. The black line is the regression line of the number of diagnoses in the 2 HCDs. The blue broken line is the 95% CI. The red line is the reference. HCD: health care department.

The concordance of new diagnoses within the 2 HCDs was subjected to analysis. Figure 2 displays the correlation coefficient within each of the Omicron subgroups, comprising vaccinated and unvaccinated adults and youths. The disparities observed between the 2 HCDs were, to some extent, attributed to the distinct protocols used by each HCD. However, these discrepancies were not deemed significant in terms of their overall impact.

## Discussion

### Principal Findings

The analysis of the long-term impact of SARS-CoV-2 Omicron infection following the acute phase was conducted using EHRs sourced from 2 distinct HCDs, comprising data from the medical records of these HCDs. Notably, vaccinated patients with Omicron infection exhibited a reduced incidence of new diagnoses, particularly among females and younger individuals, irrespective of the number of vaccine doses administered and the interval between the last dose and infection. In the case of youths, vaccination also contributed to a decrease in the incidence of new diagnoses. Additionally, vaccinated patients displayed a reduced demand for health care services compared to their unvaccinated counterparts, a pattern observed across both adult and youth populations.

Furthermore, it was observed that the overall number of new diagnoses was higher during the pre-Omicron period in comparison to the Omicron period. Specific conditions, such as depressed mood, anosmia, alopecia, dyspnea, chest pain, dysmenorrhea, myalgia, weakness, and tachycardia, were more prevalent during the pre-Omicron period, whereas conditions such as diarrhea, conjunctivitis, and low back pain exhibited higher incidence rates in the Omicron period. Despite the increase in health care resource use during the Omicron period, it remained significantly lower when compared to the pre-Omicron period. Notably, among adults, the increment in health care resource usage was more pronounced in unvaccinated patients and during the pre-Omicron period when contrasted with the vaccinated population.

To evaluate the potential impact of the viral infection itself on newly diagnosed cases and health care resource use, the inclusion of a control group becomes essential. This is because many of the newly diagnosed conditions or symptoms may not be directly caused by the virus but could instead result from factors such as stress, anxiety, or pandemic-related restrictions, affecting both individuals with and without infection to varying degrees. This study used a control group selection method based on a stringent PSM approach, which closely resembled the methodology used in a previous study conducted by our research team during the pre-Omicron pandemic waves. This earlier study used data extracted from EHRs [31] and served as a basis for comparison between the Omicron and pre-Omicron periods.

The results of this study suggest that the impact on new diagnoses and health care resource use following a less severe acute infection with the Omicron variant decreases when compared to previous pandemic waves involving other SARS-CoV-2 variants, aligning with findings from prior research [32]. However, complaints requiring health care services remain comparable, with potential differences in musculoskeletal symptoms [33]. Several factors may contribute to the reduced risk of sequelae associated with Omicron, including variances in the inherent characteristics of different SARS-CoV-2 variants in causing long-term health issues, the severity of acute infection [34], and variations in vaccination coverage and population immunity to SARS-CoV-2 [19,34,35]. Previous studies have indicated that COVID-19 vaccination and prior infections are associated with a lower risk of developing long COVID events [29,34]. Notably, the population vaccinated before the onset of acute infection exhibits fewer new diagnoses and requires fewer health care resources than its unvaccinated counterpart [36]. Currently, the effect of vaccination in reducing the risk of long COVID appears to be more prominent among women and younger individuals. Furthermore, in this study, the time elapsed between the last vaccine dose and infection did not appear to significantly impact the risk reduction. It is worth noting that individuals with a previous infection prior to the case index were excluded from the study.

The specific effects on youths have been investigated in various studies. A meta-analysis included [37] a wide range of symptoms, including fatigue, headache, loss of smell, cough, and neurological symptoms. These data were corroborated using a report from the United Kingdom, where persistent symptoms were frequently observed in English schoolchildren regardless of their SARS-CoV-2 test results. Additionally, specific symptoms, such as loss of smell and taste, were more commonly reported among those with a positive test history [38]. Furthermore, specific to youths, some studies have underscored that symptomatology can vary depending on the viral variant. This observation was reaffirmed in this study, where the impact of pre-Omicron variants was more pronounced compared to Omicron [39]. Notably, the influence of the immune response stimulated by vaccination was significant in this youth population, as differences between vaccinated and unvaccinated individuals were much more apparent than in adults.

An essential aspect that has received insufficient attention in the majority of long COVID studies pertains to the use of outpatient and hospital resources [34,40]. In this analysis, it was imperative not only to document patient numbers but also to evaluate resource use within a controlled PSM framework. The observed escalation in the health care burden, comparing cases to controls, manifested in both adults and youths, albeit with lower figures among the latter group. Across both periods, Omicron and pre-Omicron, an upsurge in visits to general practitioners, specialists, and emergency departments, as well as an increase in the necessity for hospital admissions, were noted. Although the demand for resources was more substantial in the pre-Omicron period than in the Omicron period, the demand increment in the Omicron period was also noteworthy when compared to the control group.

Several pathogenesis models have been proposed to elucidate the persistence of symptoms or the emergence of new diagnoses after SARS-CoV-2 infection. One hypothesis suggests that the persistence of the virus or a viral component [41] might exacerbate the immune response, leading to elevated levels of proinflammatory cytokines. This could potentially explain organ damage and the enduring presence of symptoms such as fatigue, headache, and olfactory dysfunction [37,42]. Furthermore, another proposed mechanism involves molecular mimicry between autoantigens and spike epitopes [42]. Nevertheless, distinguishing between functional complaints attributable to the virus and those resulting from social limitations poses a challenge in many long COVID sequelae.

### Strengths and Limitations

Both the strengths and limitations of the study merit consideration. The assessment of new diagnoses in both youth and adult cases within the general population, alongside propensity score–matched controls, facilitated the measurement of the impact of COVID-19 infection. Robust comparator data for the assessment of new diagnoses and treatments were obtained, not only through the selection of cases and controls, but also through the identification of prior diagnoses. It is important to note that the study did not encompass a clinical evaluation of the new diagnoses; however, characterizing these new diagnoses was not the primary objective of this study. Finally, it is worth acknowledging the limitations inherent in EHRs. Despite efforts to minimize these limitations, the study was restricted to patients with the necessary records for analysis.

### Conclusion

In conclusion, attention must be paid to the emergence of new diagnoses after Omicron infection, in preparation for potential future waves of SARS-CoV-2 infection. The virus’s successive mutations introduce the possibility of new waves, which may vary in terms of incidence within the general population or specific risk groups. Regardless of the severity of acute infections and the reinforced immunological status achieved through vaccination or prior infections or both, the potential repercussions for long COVID, and, subsequently, for the demands placed on the health care system, may necessitate additional resources. Vaccination plays a crucial role in mitigating the challenges faced by the health care system.

## Appendix

Multimedia Appendix 1 Supplementary tables.

## Abbreviations

CCU: critical care unit
EHR: electronic health record
HCD: health care department
ICD-10: International Classification of Diseases, 10th Revision
PSM: propensity score matching
RT-PCR: reverse transcription polymerase chain reaction

## Data Availability

The data presented in this study are available in the paper and multimedia appendices.
