# Supplementary material for: Impact of Long SARS-CoV-2 Omicron Infection on the Health Care Burden: Comparative Case-Control Study Between Omicron and Pre-Omicron Waves
Source: JMIR Public Health Surveill. 2024 Sep 3;10:e53580. doi: 10.2196/53580 (PMC11408891; doi:10.2196/53580)
Supplement: Multimedia Appendix 1 [file publichealth_v10i1e53580_app1.docx]

**Table S1.** New diagnostics (CIE-10) grouped by systems recorded in the study population ≥18 years old in the 2 centers.

|  | **OMICRON**  **PREVIOUS VACCINE** | | | | **OMICRON**  **NO PREVIOUS VACCINE** | | | | **PRE - OMICRON** | | | |
| --- | --- | --- | --- | --- | --- | --- | --- | --- | --- | --- | --- | --- |
|  | **CENTER A** | | **CENTER B** | | **CENTER A** | | **CENTER B** | | **CENTER A** | | **CENTER B** | |
|  | **Number/Incidence** | | **Number/Incidence** | | **Number/Incidence** | | **Number/Incidence** | | **Number/ Incidence** | | **Number/ Incidence** | |
| **NEUROPSYCHIATRIC** |  |  |  |  |  |  |  |  |  |  |  |  |
| F32.9-Major depressive disorder, single episode, unspecified | 71 | 0.44 | 52 | 0.41 | 8 | 0.84 | 10 | 0.51 | 72 | 0.48 | 67 | 0.48 |
| F34.1-Dysthymic disorder | 54 | 0.33 | 60 | 0.47 | 6 | 0.62 | 11 | 0.56 | 45 | 0.3 | 47 | 0.33 |
| F41.9-Anxiety disorder, unspecified | 172 | 1.45 | 165 | 1.30 | 11 | 1.54 | 33 | 1.68 | 169 | 1.53 | 151 | 1.08 |
| F43.20-Adjustment disorder, unspecified | 40 | 0.24 | 19 | 0.15 | 5 | 0.51 | 3 | 0.15 | 24 | 0.15 | 31 | 0.22 |
| F43.21-Adjustment disorder with depressed mood | 11 | 0.07 | 10 | 0.08 | 0 | 0 | 2 | 0.10 | 20 | 0.13 | 18 | 0.13 |
| G43.909-Migraine, unspecified, not intractable, without status migrainosus | 63 | 0.39 | 44 | 0.35 | 4 | 0.42 | 4 | 0.20 | 55 | 0.37 | 49 | 0.35 |
| G47.00-Insomnia, unspecified | 150 | 1.01 | 109 | 0.86 | 10 | 1.09 | 20 | 1.02 | 144 | 1.06 | 132 | 0.94 |
| R42-Dizziness and giddiness | 197 | 1.39 | 152 | 1.20 | 6 | 0.7 | 15 | 0.76 | 205 | 1.56 | 152 | 1.08 |
| R43.0-Anosmia | 4 | 0.02 | 3 | 0.02 | 0 | 0 | 1 | 0.05 | 36 | 0.23 | 46 | 0.33 |
| R51-Headache | 147 | 1.06 | 127 | 1.00 | 9 | 1.12 | 25 | 1.27 | 158 | 1.23 | 166 | 1.18 |
| **INFECTIOUS** |  |  |  |  |  |  |  |  |  |  |  |  |
| B02.9-Zoster without complications | 48 | 0.29 | 41 | 0.32 | 6 | 0.62 | 6 | 0.30 | 62 | 0.41 | 34 | 0.24 |
| J02.9-Acute pharyngitis, unspecified | 463 | 3.13 | 186 | 1.47 | 31 | 3.47 | 35 | 1.79 | 190 | 1.31 | 116 | 0.82 |
| J03.90-Acute tonsillitis, unspecified | 220 | 1.36 | 117 | 0.92 | 16 | 1.68 | 26 | 1.32 | 99 | 0.65 | 58 | 0.41 |
| R50.9-Fever, unspecified | 130 | 0.81 | 77 | 0.61 | 5 | 0.52 | 12 | 0.61 | 86 | 0.58 | 115 | 0.82 |
| **DERMATOLOGIC** |  |  |  |  |  |  |  |  |  |  |  |  |
| L29.9-Pruritus, unspecified | 60 | 0.37 | 43 | 0.34 | 5 | 0.52 | 3 | 0.15 | 44 | 0.28 | 57 | 0.4 |
| L30.9-Dermatitis, unspecified | 119 | 0.71 | 93 | 0.73 | 9 | 0.91 | 9 | 0.46 | 113 | 0.72 | 84 | 0.6 |
| L50.9-Urticaria, unspecified | 55 | 0.35 | 62 | 0.49 | 4 | 0.43 | 4 | 0.20 | 60 | 0.41 | 51 | 0.36 |
| L65.9-Nonscarring hair loss, unspecified | 51 | 0.31 | 36 | 0.28 | 3 | 0.31 | 6 | 0.30 | 104 | 0.69 | 110 | 0.78 |
| L98.9-Disorder of the skin and subcutaneous tissue, unspecified | 138 | 0.87 | 99 | 0.78 | 6 | 0.63 | 17 | 0.86 | 128 | 0.83 | 100 | 0.71 |
| **RESPIRATORY** |  |  |  |  |  |  |  |  |  |  |  |  |
| J12.89-Other viral pneumonia | 5 | 0.03 | 4 | 0.03 | 1 | 0.1 | 0 | 0.00 | 16 | 0.11 | 14 | 0.1 |
| R05-Cough | 222 | 1.5 | 177 | 1.40 | 16 | 1.79 | 23 | 1.17 | 120 | 0.87 | 117 | 0.83 |
| R06.00-Dyspnea, unspecified | 63 | 0.38 | 66 | 0.52 | 3 | 0.3 | 16 | 0.81 | 118 | 0.78 | 157 | 1.12 |
| R07.89-Other chest pain | 82 | 0.49 | 99 | 0.78 | 9 | 0.91 | 16 | 0.81 | 92 | 0.6 | 111 | 0.79 |
| R07.9-Chest pain, unspecified | 49 | 0.31 | 68 | 0.54 | 8 | 0.85 | 9 | 0.46 | 64 | 0.44 | 92 | 0.65 |
| **DIGESTIVE** |  |  |  |  |  |  |  |  |  |  |  |  |
| K21.9-Gastro-esophageal reflux disease without esophagitis | 88 | 0.56 | 49 | 0.39 | 2 | 0.21 | 8 | 0.41 | 79 | 0.55 | 50 | 0.35 |
| K30-Functional dyspepsia | 280 | 2.56 | 271 | 2.15 | 18 | 2.57 | 22 | 1.12 | 270 | 2.72 | 248 | 1.77 |
| R10.9-Unspecified abdominal pain | 184 | 1.23 | 166 | 1.31 | 13 | 1.48 | 20 | 1.02 | 170 | 1.22 | 174 | 1.24 |
| R19.7-Diarrhea, unspecified | 205 | 1.38 | 143 | 1.13 | 11 | 1.23 | 16 | 0.81 | 148 | 1.05 | 145 | 1.03 |
| **GINECOLOGIC** |  |  |  |  |  |  |  |  |  |  |  |  |
| N91.2-Amenorrhea, unspecified | 32 | 0.19 | 22 | 0.17 | 2 | 0.21 | 7 | 0.35 | 28 | 0.18 | 20 | 0.14 |
| N92.1-Excessive and frequent menstruation with irregular cycle | 34 | 0.21 | 22 | 0.17 | 1 | 0.1 | 4 | 0.20 | 25 | 0.16 | 38 | 0.27 |
| N92.6-Irregular menstruation, unspecified | 25 | 0.15 | 36 | 0.28 | 3 | 0.3 | 1 | 0.05 | 17 | 0.11 | 34 | 0.24 |
| N94.6-Dysmenorrhea, unspecified | 62 | 0.39 | 32 | 0.25 | 4 | 0.43 | 12 | 0.61 | 31 | 0.21 | 30 | 0.21 |
| **MUSCULOSKELETIC** |  |  |  |  |  |  |  |  |  |  |  |  |
| M54.5-Low back pain | 238 | 2.11 | 217 | 1.72 | 17 | 2.37 | 29 | 1.48 | 184 | 1.73 | 172 | 1.23 |
| M54.9-Dorsalgia, unspecified | 84 | 0.55 | 62 | 0.49 | 2 | 0.22 | 11 | 0.56 | 92 | 0.65 | 91 | 0.65 |
| M79.1-Myalgia | 53 | 0.32 | 72 | 0.57 | 2 | 0.2 | 13 | 0.66 | 80 | 0.52 | 91 | 0.65 |
| R53.1-Weakness | 161 | 1.01 | 85 | 0.67 | 9 | 0.95 | 19 | 0.97 | 191 | 1.3 | 174 | 1.24 |
| R53.83-Other fatigue | 34 | 0.2 | 34 | 0.27 | 3 | 0.3 | 5 | 0.25 | 46 | 0.3 | 42 | 0.3 |
| **OFTALMOLOGIC** |  |  |  |  |  |  |  |  |  |  |  |  |
| H10.9-Unspecified conjunctivitis | 155 | 0.93 | 105 | 0.83 | 7 | 0.7 | 12 | 0.61 | 69 | 0.44 | 75 | 0.53 |
| **HEMATOLOGIC** |  |  |  |  |  |  |  |  |  |  |  |  |
| D64.9-Anemia, unspecified | 74 | 0.48 | 66 | 0.52 | 3 | 0.32 | 13 | 0.66 | 69 | 0.48 | 90 | 0.64 |
| **CARDIOVASCULAR** |  |  |  |  |  |  |  |  |  |  |  |  |
| R00.0-Tachycardia, unspecified | 42 | 0.25 | 23 | 0.18 | 3 | 0.3 | 7 | 0.35 | 51 | 0.33 | 48 | 0.34 |
| R00.2-Palpitations | 29 | 0.17 | 45 | 0.35 | 5 | 0.51 | 6 | 0.30 | 42 | 0.27 | 52 | 0.37 |
| **ORAL_CAVITY** |  |  |  |  |  |  |  |  |  |  |  |  |
| K12.0-Recurrent oral aphthae | 41 | 0.25 | 26 | 0.20 | 2 | 0.2 | 4 | 0.20 | 27 | 0.18 | 32 | 0.23 |

**Table S2.** New diagnostics (CIE-10) grouped by systems recorded in the study population <18 years old in the 2 centers.

|  | **OMICRON**  **PREVIOUS VACCINE** | | | | **OMICRON**  **NO PREVIOUS VACCINE** | | | | **PRE - OMICRON** | | | |
| --- | --- | --- | --- | --- | --- | --- | --- | --- | --- | --- | --- | --- |
|  | **CENTER A** | | **CENTER B** | | **CENTER A** | | **CENTER B** | | **CENTER A** | | **CENTER B** | |
|  | **Number/Incidence** | | **Number/Incidence** | | **Number/Incidence** | | **Number/Incidence** | | **Number/Incidence** | | **Number/Incidence** | |
| **NEUROPSYCHIATRIC** |  |  |  |  |  |  |  |  |  |  |  |  |
| F32.9-Major depressive disorder, single episode, unspecified | 2 | 0.08 | 5 | 0.22 | 1 | 0.06 | 0 | 0.00 | 6 | 0.15 | 5 | 0.14 |
| F34.1-Dysthymic disorder | 4 | 0.15 | 2 | 0.09 | 0 | 0 | 0 | 0.00 | 4 | 0.10 | 1 | 0.03 |
| F41.9-Anxiety disorder, unspecified | 25 | 0.99 | 18 | 0.81 | 2 | 0.11 | 3 | 0.19 | 34 | 0.91 | 28 | 0.78 |
| F43.20-Adjustment disorder, unspecified | 1 | 0.04 | 2 | 0.09 | 0 | 0 | 0 | 0.00 | 2 | 0.05 | 6 | 0.17 |
| F43.21-Adjustment disorder with depressed mood | 1 | 0.04 | 0 | 0 | 0 | 0 | 0 | 0.00 | 2 | 0.05 | 3 | 0.08 |
| G43.909-Migraine, unspecified, not intractable, without status migrainosus | 6 | 0.23 | 5 | 0.22 | 1 | 0.06 | 2 | 0.13 | 7 | 0.18 | 11 | 0.31 |
| G47.00-Insomnia, unspecified | 5 | 0.19 | 6 | 0.27 | 1 | 0.06 | 1 | 0.06 | 2 | 0.05 | 7 | 0.2 |
| R42-Dizziness and giddiness | 18 | 0.71 | 29 | 1.30 | 7 | 0.39 | 4 | 0.26 | 22 | 0.59 | 30 | 0.84 |
| R43.0-Anosmia | 0 | 0 | 0 | 0 | 0 | 0 | 0 | 0.00 | 3 | 0.08 | 5 | 0.14 |
| R51-Headache | 33 | 1.55 | 40 | 1.80 | 18 | 1.07 | 16 | 1.04 | 47 | 1.41 | 47 | 1.32 |
| **INFECTIOUS** |  |  |  |  |  |  |  |  |  |  |  |  |
| B02.9-Zoster without complications | 3 | 0.11 | 0 | 0.00 | 1 | 0.06 | 2 | 0.09 | 3 | 0.08 | 2 | 0.06 |
| J02.9-Acute pharyngitis, unspecified | 109 | 4.91 | 63 | 2.85 | 89 | 6.03 | 79 | 5.26 | 83 | 2.42 | 30 | 0.84 |
| J03.90-Acute tonsillitis, unspecified | 70 | 2.97 | 30 | 1.35 | 99 | 6.54 | 59 | 3.90 | 76 | 2.14 | 44 | 1.23 |
| R50.9-Fever, unspecified | 61 | 3.05 | 41 | 1.85 | 103 | 11.55 | 83 | 5.55 | 79 | 2.71 | 75 | 2.11 |
| **DERMATOLOGIC** |  |  |  |  |  |  |  |  |  |  |  |  |
| L29.9-Pruritus, unspecified | 2 | 0.08 | 6 | 0.27 | 4 | 0.23 | 4 | 0.26 | 5 | 0.13 | 6 | 0.17 |
| L30.9-Dermatitis, unspecified | 15 | 0.58 | 12 | 0.54 | 22 | 1.31 | 11 | 0.72 | 31 | 0.81 | 22 | 0.61 |
| L50.9-Urticaria, unspecified | 16 | 0.68 | 15 | 0.67 | 17 | 1.05 | 15 | 0.98 | 24 | 0.68 | 16 | 0.45 |
| L65.9-Nonscarring hair loss, unspecified | 2 | 0.08 | 4 | 0.18 | 2 | 0.11 | 1 | 0.06 | 5 | 0.13 | 7 | 0.2 |
| L98.9-Disorder of the skin and subcutaneous tissue, unspecified | 13 | 0.51 | 6 | 0.27 | 9 | 0.51 | 9 | 0.59 | 25 | 0.66 | 21 | 0.59 |
| **RESPIRATORY** |  |  |  |  |  |  |  |  |  |  |  |  |
| J12.89-Other viral pneumonia | 0 | 0 | 0 | 0.00 | 0 | 0 | 0 | 0.00 |  | 0.00 | 0 | 0 |
| R05-Cough | 34 | 1.72 | 28 | 1.26 | 59 | 4.57 | 46 | 3.03 | 49 | 1.64 | 47 | 1.32 |
| R06.00-Dyspnea, unspecified | 0 | 0 | 3 | 0.13 | 0 | 0 | 1 | 0.06 | 3 | 0.08 | 9 | 0.25 |
| R07.89-Other chest pain | 5 | 0.19 | 6 | 0.27 | 3 | 0.17 | 2 | 0.13 | 6 | 0.15 | 11 | 0.31 |
| R07.9-Chest pain, unspecified | 9 | 0.35 | 9 | 0.40 | 3 | 0.17 | 4 | 0.26 | 10 | 0.26 | 10 | 0.28 |
| **DIGESTIVE** |  |  |  |  |  |  |  |  |  |  |  |  |
| K21.9-Gastro-esophageal reflux disease without esophagitis | 3 | 0.11 | 3 | 0.13 | 2 | 0.11 | 2 | 0.13 | 4 | 0.10 | 6 | 0.17 |
| K30-Functional dyspepsia | 11 | 0.43 | 9 | 0.40 | 1 | 0.06 | 2 | 0.13 | 17 | 0.43 | 25 | 0.7 |
| R10.9-Unspecified abdominal pain | 38 | 1.94 | 57 | 2.58 | 37 | 2.43 | 41 | 2.69 | 65 | 2.09 | 72 | 2.03 |
| R19.7-Diarrhea, unspecified | 19 | 0.87 | 13 | 0.58 | 30 | 2.27 | 26 | 1.70 | 41 | 1.32 | 41 | 1.15 |
| **GINECOLOGIC** |  |  |  |  |  |  |  |  |  |  |  |  |
| N91.2-Amenorrhea, unspecified | 2 | 0.08 | 4 | 0.18 | 1 | 0.06 | 0 | 0.00 | 5 | 0.13 | 4 | 0.11 |
| N92.1-Excessive and frequent menstruation with irregular cycle | 0 | 0 | 1 | 0.04 | 1 | 0.06 | 0 | 0.00 | 2 | 0.05 | 3 | 0.08 |
| N92.6-Irregular menstruation, unspecified | 6 | 0.23 | 4 | 0.18 | 1 | 0.06 | 1 | 0.06 | 8 | 0.21 | 9 | 0.25 |
| N94.6-Dysmenorrhea, unspecified | 22 | 0.85 | 18 | 0.81 | 1 | 0.06 | 4 | 0.26 | 19 | 0.50 | 21 | 0.59 |
| **MUSCULOSKELETIC** |  |  |  |  |  |  |  |  |  |  |  |  |
| M54.5-Low back pain | 13 | 0.5 | 9 | 0.40 | 0 | 0 | 3 | 0.19 | 19 | 0.50 | 15 | 0.42 |
| M54.9-Dorsalgia, unspecified | 13 | 0.5 | 5 | 0.22 | 4 | 0.22 | 2 | 0.13 | 11 | 0.29 | 8 | 0.22 |
| M79.1-Myalgia | 1 | 0.04 | 12 | 0.54 | 4 | 0.23 | 4 | 0.26 | 9 | 0.24 | 10 | 0.28 |
| R53.1-Weakness | 14 | 0.54 | 16 | 0.72 | 3 | 0.17 | 6 | 0.39 | 22 | 0.58 | 17 | 0.47 |
| R53.83-Other fatigue | 4 | 0.15 | 4 | 0.18 | 1 | 0.06 | 1 | 0.06 | 11 | 0.28 | 5 | 0.14 |
| **OFTALMOLOGIC** |  |  |  |  |  |  |  |  |  |  |  |  |
| H10.9-Unspecified conjunctivitis | 18 | 0.69 | 16 | 0.72 | 61 | 3.71 | 44 | 2.89 | 19 | 0.50 | 24 | 0.67 |
| **HEMATOLOGIC** |  |  |  |  |  |  |  |  |  |  |  |  |
| D64.9-Anemia, unspecified | 4 | 0.15 | 6 | 0.22 | 1 | 0.06 | 4 | 0.26 | 5 | 0.13 | 11 | 0.31 |
| **CARDIOVASCULAR** |  |  |  |  |  |  |  |  |  |  |  |  |
| R00.0-Tachycardia, unspecified | 3 | 0.11 | 4 | 0.18 | 1 | 0.06 | 2 | 0.13 | 2 | 0.05 | 3 | 0.06 |
| R00.2-Palpitations | 3 | 0.11 | 2 | 0.09 | 0 | 0 | 0 | 0.00 | 5 | 0.13 | 3 | 0.08 |
| **ORAL_CAVITY** |  |  |  |  |  |  |  |  |  |  |  |  |
| K12.0-Recurrent oral aphthae | 6 | 0.24 | 5 | 0.22 | 40 | 2.44 | 25 | 1.64 | 9 | 0.25 | 20 | 0.56 |

**Table S3.** New diagnostics (CIE-10) grouped by systems recorded in patients and controls of the study population ≥18 years old in the 2 centers.

|  | **OMICRON**  **PREVIOUS VACCINE** | | | | | | **OMICRON**  **NO VACCINE** | | | | | |
| --- | --- | --- | --- | --- | --- | --- | --- | --- | --- | --- | --- | --- |
|  | **CENTER A** | | | **CENTER B** | | | **CENTER A** | | | **CENTER B** | | |
|  | **CASE** | **CONTROL** | **%** | **CASE** | **CONTROL** | **%** | **CASE** | **CONTROL** | **%** | **CASE** | **CONTROL** | **%** |
| **NEUROPSYCHIATRIC** |  |  |  |  |  |  |  |  |  |  |  |  |
| F32.9-Major depressive disorder, single episode, unspecified | 71 | 61 | 14% | 72 | 55 | 24% | 8 | 2 | 75% | 10 | 6 | 40% |
| F34.1-Dysthymic disorder | 54 | 37 | 31% | 66 | 37 | 44% | 6 | 1 | 83% | 15 | 4 | 73% |
| F41.9-Anxiety disorder, unspecified | 172 | 137 | 20% | 176 | 124 | 30% | 11 | 5 | 55% | 35 | 22 | 37% |
| F43.20-Adjustment disorder, unspecified | 40 | 28 | 30% | 25 | 41 | -64% | 5 | 2 | 60% | 3 | 5 | -67% |
| F43.21-Adjustment disorder with depressed mood | 11 | 11 | 0% | 10 | 36 | -260% | 0 | 0 |  | 2 | 2 | 0% |
| G43.909-Migraine, unspecified, not intractable, without status migrainosus | 63 | 38 | 40% | 44 | 35 | 20% | 4 | 1 | 75% | 4 | 7 | -75% |
| G47.00-Insomnia, unspecified | 150 | 94 | 37% | 113 | 76 | 33% | 10 | 4 | 60% | 20 | 15 | 25% |
| R42-Dizziness and giddiness | 197 | 149 | 24% | 169 | 135 | 20% | 6 | 4 | 33% | 17 | 11 | 35% |
| R43.0-Anosmia | 4 | 3 | 25% | 4 | 3 | 25% | 0 | 0 | -- | 1 | 0 | 100% |
| R51-Headache | 147 | 105 | 29% | 133 | 97 | 27% | 9 | 4 | 56% | 25 | 14 | 44% |
| **INFECTIOUS** |  |  |  |  |  |  |  |  |  |  |  |  |
| B02.9-Zoster without complications | 48 | 31 | 35% | 48 | 33 | 31% | 6 | 1 | 83% | 6 | 1 | 83% |
| J02.9-Acute pharyngitis, unspecified | 463 | 341 | 26% | 193 | 187 | 3% | 31 | 10 | 68% | 37 | 14 | 62% |
| J03.90-Acute tonsillitis, unspecified | 220 | 137 | 38% | 122 | 72 | 41% | 16 | 4 | 75% | 27 | 11 | 59% |
| R50.9-Fever, unspecified | 130 | 74 | 43% | 96 | 122 | -27% | 5 | 6 | -20% | 24 | 7 | 71% |
| **DERMATOLOGIC** |  |  |  |  |  |  |  |  |  |  |  |  |
| L29.9-Pruritus, unspecified | 60 | 44 | 27% | 47 | 39 | 17% | 5 | 2 | 60% | 3 | 1 | 67% |
| L30.9-Dermatitis, unspecified | 119 | 109 | 8% | 113 | 92 | 19% | 9 | 1 | 89% | 9 | 9 | 0% |
| L50.9-Urticaria, unspecified | 55 | 72 | -31% | 66 | 57 | 14% | 4 | 2 | 50% | 4 | 6 | -50% |
| L65.9-Nonscarring hair loss, unspecified | 51 | 27 | 47% | 37 | 29 | 22% | 3 | 1 | 67% | 6 | 6 | 0% |
| L98.9-Disorder of the skin and subcutaneous tissue, unspecified | 138 | 93 | 33% | 99 | 70 | 29% | 6 | 3 | 50% | 18 | 4 | 78% |
| **RESPIRATORY** |  |  |  |  |  |  |  |  |  |  |  |  |
| J12.89-Other viral pneumonia | 5 | 0 | 100% | 10 | 0 | 100% | 1 | 0 | 100% | 0 | 0 |  |
| R05-Cough | 222 | 159 | 28% | 190 | 123 | 35% | 16 | 7 | 56% | 24 | 11 | 54% |
| R06.00-Dyspnea, unspecified | 63 | 44 | 30% | 73 | 44 | 40% | 3 | 1 | 67% | 21 | 8 | 62% |
| R07.89-Other chest pain | 82 | 50 | 39% | 117 | 77 | 34% | 9 | 0 | 100% | 16 | 12 | 25% |
| R07.9-Chest pain, unspecified | 49 | 35 | 29% | 79 | 47 | 41% | 8 | 1 | 88% | 9 | 7 | 22% |
| **DIGESTIVE** |  |  |  |  |  |  |  |  |  |  |  |  |
| K21.9-Gastro-esophageal reflux disease without esophagitis | 88 | 69 | 22% | 53 | 44 | 17% | 2 | 1 | 50% | 11 | 12 | -9% |
| K30-Functional dyspepsia | 280 | 217 | 23% | 283 | 186 | 34% | 18 | 13 | 28% | 22 | 23 | -5% |
| R10.9-Unspecified abdominal pain | 184 | 119 | 35% | 207 | 152 | 27% | 13 | 6 | 54% | 24 | 29 | -21% |
| R19.7-Diarrhea, unspecified | 205 | 116 | 43% | 177 | 109 | 38% | 11 | 2 | 82% | 16 | 8 | 50% |
| **GINECOLOGIC** |  |  |  |  |  |  |  |  |  |  |  |  |
| N91.2-Amenorrhea, unspecified | 32 | 21 | 34% | 26 | 23 | 12% | 2 | 1 | 50% | 7 | 1 | 86% |
| N92.1-Excessive and frequent menstruation with irregular cycle | 34 | 22 | 35% | 31 | 35 | -13% | 1 | 0 | 100% | 4 | 8 | -100% |
| N92.6-Irregular menstruation, unspecified | 25 | 31 | -24% | 39 | 27 | 31% | 3 | 1 | 67% | 1 | 3 | -200% |
| N94.6-Dysmenorrhea, unspecified | 62 | 42 | 32% | 40 | 36 | 10% | 4 | 2 | 50% | 12 | 6 | 50% |
| **MUSCULOSKELETIC** |  |  |  |  |  |  |  |  |  |  |  |  |
| M54.5-Low back pain | 238 | 176 | 26% | 237 | 207 | 13% | 17 | 10 | 41% | 38 | 20 | 47% |
| M54.9-Dorsalgia, unspecified | 84 | 49 | 42% | 64 | 65 | -2% | 2 | 1 | 50% | 12 | 5 | 58% |
| M79.1-Myalgia | 53 | 44 | 17% | 77 | 66 | 14% | 2 | 2 | 0% | 13 | 6 | 54% |
| R53.1-Weakness | 161 | 109 | 32% | 93 | 66 | 29% | 9 | 5 | 44% | 20 | 11 | 45% |
| R53.83-Other fatigue | 34 | 28 | 18% | 34 | 12 | 65% | 3 | 0 | 100% | 5 | 1 | 80% |
| **OFTALMOLOGIC** |  |  |  |  |  |  |  |  |  |  |  |  |
| H10.9-Unspecified conjunctivitis | 155 | 88 | 43% | 106 | 76 | 28% | 7 | 4 | 43% | 12 | 9 | 25% |
| **HEMATOLOGIC** |  |  |  |  |  |  |  |  |  |  |  |  |
| D64.9-Anemia, unspecified | 74 | 62 | 16% | 86 | 77 | 10% | 3 | 3 | 0% | 36 | 12 | 67% |
| **CARDIOVASCULAR** |  |  |  |  |  |  |  |  |  |  |  |  |
| R00.0-Tachycardia, unspecified | 42 | 25 | 40% | 25 | 17 | 32% | 3 | 1 | 67% | 8 | 1 | 88% |
| R00.2-Palpitations | 29 | 25 | 14% | 58 | 21 | 64% | 5 | 0 | 100% | 8 | 3 | 63% |
| **ORAL_CAVITY** |  |  |  |  |  |  |  |  |  |  |  |  |
| K12.0-Recurrent oral aphthae | 41 | 21 | 49% | 26 | 10 | 62% | 2 | 0 | 100% | 5 | 1 | 80% |

**Table S4.** New diagnostics (CIE-10) grouped by systems recorded in patients and controls of the study population <18 years old in the 2 centers.

|  | **OMICRON**  **PREVIOUS VACCINE** | | | | | | | **OMICRON**  **NO VACCINE** | | | | | | | |
| --- | --- | --- | --- | --- | --- | --- | --- | --- | --- | --- | --- | --- | --- | --- | --- |
|  | **CENTER A** | | | | **CENTER B** | | | **CENTER A** | | | | **CENTER B** | | | |
|  | **CASE** | **CONTROL** |  | | **CASE** | **CONTROL** |  | **CASE** | **CONTROL** | |  | **CASE** | | **CONTROL** |  |
| **NEUROPSYCHIATRIC** |  |  |  | |  |  |  |  |  | |  |  | |  |  |
| F32.9-Major depressive disorder, single episode, unspecified | 2 | 6 | -200% | | 6 | 7 | -17% | 1 | 0 | 100% | | 0 | | 0 |  |
| F34.1-Dysthymic disorder | 4 | 1 | 75% | | 2 | 1 | 50% | 0 | 0 | -- | | 0 | | 0 | -- |
| F41.9-Anxiety disorder, unspecified | 25 | 13 | 48% | | 21 | 28 | -33% | 2 | 5 | -150% | | 4 | | 7 | -75% |
| F43.20-Adjustment disorder, unspecified | 1 | 0 | 100% | | 2 | 3 | -50% | 0 | 0 | -- | | 0 | | 0 | -- |
| F43.21-Adjustment disorder with depressed mood | 1 | 2 | -100% | | 0 | 1 | -- | 0 | 0 | -- | | 0 | | 0 | -- |
| G43.909-Migraine, unspecified, not intractable, without status migrainosus | 6 | 2 | 67% | | 6 | 10 | -67% | 1 | 1 | 0% | | 2 | | 3 | -50% |
| G47.00-Insomnia, unspecified | 5 | 1 | 80% | | 7 | 4 | 43% | 1 | 2 | -100% | | 1 | | 0 | 100% |
| R42-Dizziness and giddiness | 18 | 15 | 17% | | 29 | 11 | 62% | 7 | 5 | 29% | | 4 | | 1 | 75% |
| R43.0-Anosmia | 0 | 0 | -- | | 0 | 0 | -- | 0 | 0 | -- | | 0 | | 0 | -- |
| R51-Headache | 33 | 27 | 18% | | 40 | 27 | 33% | 18 | 13 | 28% | | 18 | | 11 | 39% |
| **INFECTIOUS** |  |  | |  |  |  |  |  |  |  | |  | |  |  |
| B02.9-Zoster without complications | 3 | 1 | | 67% | 0 | 1 | -- | 1 | 0 | 100% | | 2 | | 0 | 100% |
| J02.9-Acute pharyngitis, unspecified | 109 | 83 | | 24% | 66 | 46 | 30% | 89 | 62 | 30% | | 93 | | 47 | 49% |
| J03.90-Acute tonsillitis, unspecified | 70 | 55 | | 21% | 32 | 30 | 6% | 99 | 69 | 30% | | 65 | | 59 | 9% |
| R50.9-Fever, unspecified | 61 | 50 | | 18% | 49 | 54 | -10% | 103 | 91 | 12% | | 98 | | 67 | 32% |
| **DERMATOLOGIC** |  |  | |  |  |  |  |  |  |  | |  | |  |  |
| L29.9-Pruritus, unspecified | 2 | 3 | | -50% | 6 | 6 | 0% | 4 | 3 | 25% | | 4 | | 3 | 25% |
| L30.9-Dermatitis, unspecified | 15 | 9 | | 40% | 13 | 14 | -8% | 22 | 23 | -5% | | 11 | | 9 | 18% |
| L50.9-Urticaria, unspecified | 16 | 9 | | 44% | 16 | 9 | 44% | 17 | 10 | 41% | | 15 | | 8 | 47% |
| L65.9-Nonscarring hair loss, unspecified | 2 | 2 | | 0% | 4 | 4 | 0% | 2 | 0 | 100% | | 1 | | 1 | 0% |
| L98.9-Disorder of the skin and subcutaneous tissue, unspecified | 13 | 10 | | 23% | 6 | 13 | -117% | 9 | 6 | 33% | | 9 | | 4 | 56% |
| **RESPIRATORY** |  |  | |  |  |  |  |  |  |  | |  | |  |  |
| J12.89-Other viral pneumonia | 0 | 0 | | -- | 0 | 0 | -- | 0 | 0 | -- | | 0 | | 1 | -- |
| R05-Cough | 34 | 28 | | 18% | 28 | 22 | 21% | 59 | 43 | 27% | | 48 | | 28 | 42% |
| R06.00-Dyspnea, unspecified | 0 | 0 | | -- | 3 | 2 | 33% | 0 | 1 | -- | | 1 | | 0 | 100% |
| R07.89-Other chest pain | 5 | 4 | | 20% | 7 | 5 | 29% | 3 | 2 | 33% | | 2 | | 0 | 100% |
| R07.9-Chest pain, unspecified | 9 | 1 | | 89% | 10 | 8 | 20% | 3 | 3 | 0% | | 4 | | 4 | 0% |
| **DIGESTIVE** |  |  | |  |  |  |  |  |  |  | |  | |  |  |
| K21.9-Gastro-esophageal reflux disease without esophagitis | 3 | 1 | | 67% | 4 | 1 | 75% | 2 | 3 | -50% | | 4 | | 3 | 25% |
| K30-Functional dyspepsia | 11 | 11 | | 0% | 9 | 10 | -11% | 1 | 0 | 100% | | 2 | | 4 | -100% |
| R10.9-Unspecified abdominal pain | 38 | 33 | | 13% | 70 | 57 | 19% | 37 | 23 | 38% | | 49 | | 52 | -6% |
| R19.7-Diarrhea, unspecified | 19 | 15 | | 21% | 14 | 9 | 36% | 30 | 27 | 10% | | 30 | | 17 | 43% |
| **GINECOLOGIC** |  |  | |  |  |  |  |  |  |  | |  | |  |  |
| N91.2-Amenorrhea, unspecified | 2 | 3 | | -50% | 4 | 2 | 50% | 1 | 0 | 100% | | 0 | | 0 | -- |
| N92.1-Excessive and frequent menstruation with irregular cycle | 0 | 2 | | -- | 1 | 2 | -100% | 1 | 0 | 100% | | 0 | | 0 | -- |
| N92.6-Irregular menstruation, unspecified | 6 | 4 | | 33% | 4 | 3 | 25% | 1 | 0 | 100% | | 1 | | 0 | 100% |
| N94.6-Dysmenorrhea, unspecified | 22 | 22 | | 0% | 18 | 17 | 6% | 1 | 0 | 100% | | 4 | | 3 | 25% |
| **MUSCULOSKELETIC** |  |  | |  |  |  |  |  |  |  | |  | |  |  |
| M54.5-Low back pain | 13 | 6 | | 54% | 10 | 5 | 50% | 0 | 1 |  | | 3 | | 1 | 67% |
| M54.9-Dorsalgia, unspecified | 13 | 4 | | 69% | 5 | 6 | -20% | 4 | 3 | 25% | | 2 | | 1 | 50% |
| M79.1-Myalgia | 1 | 9 | | -800% | 12 | 6 | 50% | 4 | 2 | 50% | | 4 | | 2 | 50% |
| R53.1-Weakness | 14 | 10 | | 29% | 18 | 9 | 50% | 3 | 2 | 33% | | 6 | | 3 | 50% |
| R53.83-Other fatigue | 4 | 4 | | 0% | 4 | 2 | 50% | 1 | 2 | -100% | | 1 | | 2 | -100% |
| **OFTALMOLOGIC** |  |  | |  |  |  |  |  |  |  | |  | |  |  |
| H10.9-Unspecified conjunctivitis | 18 | 27 | | -50% | 16 | 20 | -25% | 61 | 47 | 23% | | | 47 | 47 | 0% |
| **HEMATOLOGIC** |  |  | |  |  |  |  |  |  |  | | |  |  |  |
| D64.9-Anemia, unspecified | 4 | 4 | | 0% | 6 | 9 | -50% | 1 | 4 | -300% | | | 4 | 0 | 100% |
| **CARDIOVASCULAR** |  |  | |  |  |  |  |  |  |  | | |  |  |  |
| R00.0-Tachycardia, unspecified | 3 | 1 | | 67% | 4 | 0 | 100% | 1 | 1 | 0% | | | 2 | 0 | 100% |
| R00.2-Palpitations | 3 | 2 | | 33% | 4 | 2 | 50% | 0 | 1 | -- | | | 0 | 0 | -- |
| **ORAL_CAVITY** |  |  | |  |  |  |  |  |  |  | | |  |  |  |
| K12.0-Recurrent oral aphthae | 6 | 11 | | -83% | 5 | 8 | -60% | 40 | 34 | 15% | | | 29 | 18 | 38% |

**Table S5.** P values of the differences in incidence between Omicron vaccinated or not vaccinated and pre-Omicron in the study population ≥18 and <18 years old. Diagnostics were (CIE-10) grouped by systems.

|  | **Age >17 year old** | | **Age < 18 year old** | |
| --- | --- | --- | --- | --- |
|  | **Vaccine**  **Yes vs No** | **Omicron vs Pre-Omicron** | **Vaccine**  **Yes vs No** | **Omicron vs Pre-Omicron** |
| **NEUROPSYCHIATRIC** |  |  |  |  |
| F32.9-Major depressive disorder, single episode, unspecified | 0,145 | 0,55 | 0,154 | 0,495 |
| F34.1-Dysthymic disorder | 0,131 | 0,06 | 0,088 | 1 |
| F41.9-Anxiety disorder, unspecified | 0,263 | 0,227 | <0.001 | 0,069 |
| F43.20-Adjustment disorder, unspecified | 0,395 | 0,526 | 0,278 | 0,137 |
| F43.21-Adjustment disorder with depressed mood | 1 | 0,03 | 1 | 0,12 |
| G43.909-Migraine, unspecified, not intractable, without status migrainosus | 0,518 | 1 | 0,184 | 0,38 |
| G47.00-Insomnia, unspecified | 0,615 | 0,556 | 0,091 | 0,53 |
| R42-Dizziness and giddiness | 0,01 | 0,504 | <0.001 | 1 |
| R43.0-Anosmia | 0,494 | <0.001 | 1 | 0,003 |
| R51-Headache | 0,33 | 0,094 | 0,025 | 0,832 |
| **INFECTIOUS** |  |  |  |  |
| B02.9-Zoster without complications | 0,303 | 0,776 | 1 | 1 |
| J02.9-Acute pharyngitis, unspecified | 0,949 | <0.001 | <0.001 | <0.001 |
| J03.90-Acute tonsillitis, unspecified | 0,212 | <0.001 | <0.001 | <0.001 |
| R50.9-Fever, unspecified | 0,488 | 0,884 | <0.001 | <0.001 |
| **DERMATOLOGIC** |  |  |  |  |
| L29.9-Pruritus, unspecified | 0,621 | 0,837 | 0,459 | 0,564 |
| L30.9-Dermatitis, unspecified | 0,567 | 0,498 | 0,024 | 0,851 |
| L50.9-Urticaria, unspecified | 0,353 | 0,795 | 0,123 | 0,076 |
| L65.9-Nonscarring hair loss, unspecified | 0,86 | <0.001 | 0,744 | 0,392 |
| L98.9-Disorder of the skin and subcutaneous tissue, unspecified | 0,915 | 0,469 | 0,403 | 0,155 |
| **RESPIRATORY** |  |  |  |  |
| J12.89-Other viral pneumonia | 1 | <0.001 | 1 | 1 |
| R05-Cough | 0,806 | <0.001 | <0.001 | <0.001 |
| R06.00-Dyspnea, unspecified | 0,152 | <0.001 | 0,653 | 0,043 |
| R07.89-Other chest pain | 0,145 | 0,428 | 0,612 | 0,73 |
| R07.9-Chest pain, unspecified | 0,178 | 0,04 | 0,226 | 0,766 |
| **DIGESTIVE** |  |  |  |  |
| K21.9-Gastro-esophageal reflux disease without esophagitis | 0,393 | 0,763 | 1 | 0,827 |
| K30-Functional dyspepsia | 0,005 | 0,489 | 0,009 | 0,012 |
| R10.9-Unspecified abdominal pain | 0,725 | 0,767 | 0,443 | 0,191 |
| R19.7-Diarrhea, unspecified | 0,157 | 0,03 | <0.001 | 0,939 |
| **GINECOLOGIC** |  |  |  |  |
| N91.2-Amenorrhea, unspecified | 0,18 | 0,343 | 0,253 | 0,621 |
| N92.1-Excessive and frequent menstruation with irregular cycle | 1 | 0,59 | 1 | 0,283 |
| N92.6-Irregular menstruation, unspecified | 0,521 | 0,457 | 0,141 | 0,269 |
| N94.6-Dysmenorrhea, unspecified | 0,067 | 0,001 | <0.001 | 0,914 |
| **MUSCULOSKELETIC** |  |  |  |  |
| M54.5-Low back pain | 0,553 | <0.001 | 0,004 | 0,152 |
| M54.9-Dorsalgia, unspecified | 0,784 | 0,035 | 0,146 | 0,761 |
| M79.1-Myalgia | 0,557 | 0,011 | 0,829 | 1 |
| R53.1-Weakness | 0,532 | <0.001 | 0,033 | 0,734 |
| R53.83-Other fatigue | 0,688 | 0,118 | 0,333 | 0,176 |
| **OFTALMOLOGIC** |  |  |  |  |
| H10.9-Unspecified conjunctivitis | 0,179 | <0.001 | <0.001 | <0.001 |
| **HEMATOLOGIC** |  |  |  |  |
| D64.9-Anemia, unspecified | 0,68 | 0,367 | 1 | 0,592 |
| **CARDIOVASCULAR** |  |  |  |  |
| R00.0-Tachycardia, unspecified | 0,225 | 0,015 | 0,749 | 0,196 |
| R00.2-Palpitations | 0,252 | 0,203 | 0,164 | 0,411 |
| **ORAL_CAVITY** |  |  |  |  |
| K12.0-Recurrent oral aphthae | 1 | 0,543 | <0.001 | <0.001 |
